# Shed Syndecans (1–3), ELA-32, BDNF, NLR, and hs-CRP in Parkinson’s Disease: Appropriate Diagnostic and Prognostic Biomarkers When Combined in a Unique Panel

**DOI:** 10.3390/ijms26104503

**Published:** 2025-05-08

**Authors:** Carmela Rita Balistreri, Daniele Magro, Letizia Scola, Paolo Aridon, Paolo Ragonese, Felipe Augusto Dos Santos Mendes, Giuseppe Schirò, Marco D’Amelio

**Affiliations:** 1 Cellular, Molecular and Clinical Pathological Laboratory, Department of Biomedicine, Neuroscience and Advanced Diagnostics (Bi.N.D.), University of Palermo, 90134 Palermo, Italy; daniele.magro@unipa.it (D.M.); letizia.scola@unipa.it (L.S.); 2 Department of Biomedicine, Neuroscience and Advanced Diagnostics, University of Palermo, 90129 Palermo, Italy; paolo.aridon@unipa.it (P.A.); paolo.ragonese@unipa.it (P.R.); giuseppeschiro1994@gmail.com (G.S.); marco.damelio@unipa.it (M.D.); 3 Graduate Program in Rehabilitation Sciences, University of Brasília, Brasília 72220-275, Brazil; mendesf.fm@gmail.com; 4 Multiple Sclerosis Center, Foundation Institute G. Giglio, Cefalù, 90015 Palermo, Italy

**Keywords:** inflammatory molecules (i.e., hs-CRP) and their ratios (i.e., NLR), glycocalyx shedding products, ELA peptides, BDNF, idiopathic Parkinson’s disease (PD), risk factors, diagnostic biomarkers

## Abstract

Currently, the management of Parkinson’s disease (PD), the second most common neurodegenerative disease, is challenging due to the lack of consensus on blood biomarkers for diagnostic, prognostic, and outcome purposes. The identification of specific and sensitive biomarkers could contribute to an early diagnosis and, consequently, facilitate management and improve prognosis. Several molecules are emerging as potential biomarkers, although current data seem preliminary and need further validation. Further, their combination in a panel seems to strengthen their diagnostic power, allowing them to identify PD cases with greater specificity and sensitivity. In this study, we evaluated the association of a panel of molecules, including shed syndecans, ELA peptides, CD141, VEGF, BDNF, and systemic inflammatory indices, in 30 PD cases and 30 matched healthy controls. Significant differences in the systemic levels of all the molecules studied were detected in the PD group when compared to the healthy participants. Univariate and multivariate regression analyses, as well as correlations with clinical indicators, including PD severity expressed by the Hoehn and Yahr (H&Y) scale, highlighted the key role of the studied molecules as independent risk factors. Finally, the use of receiver operating characteristic (ROC) curves demonstrated the diagnostic value of hs-CRP, NLR, BDNF, shed syndecans (1–3), and ELA-32 in PD. Interestingly, their diagnostic performance significantly improved when combined in a panel. Overall, our results suggest that hs-CRP, NLR, BDNF, shed syndecans (1–3), and ELA-32 are significantly associated with PD and could likely serve as appropriate diagnostic and prognostic biomarkers, especially if combined in a panel.

## 1. Introduction

Parkinson’s disease (PD), as well as parkinsonism, is a progressive neurodegenerative disease (ND), whose management is very complex [1]. The identification of specific and sensitive biomarkers could contribute to an early diagnosis, facilitating its management and improving its prognosis [2,3,4]. On the other hand, α-synuclein, phosphorylated at serine 129 (pS129-α-syn), has recently been shown to have moderate potential as a diagnostic PD biomarker, and especially as a differential biomarker [5,6]. Therefore, its diagnostic value remains limited and, rather, requires its combination with other biomarkers to increase diagnostic accuracy and prognostic value [7]. Recently, shed syndecans (belonging to the type I transmembrane heparan sulfate proteoglycan family, composed of four members, i.e., SDC-1, SDC-2, SDC-3, and SDC-4) and other dysfunction-related molecules of the endothelial glycocalyx (eGCX) of endothelial cells (ECs) of the blood–brain barrier (BBB) and brain capillary wall (i.e., Elabela (ELA)-54, ELA-21, and ELA-32, thrombomodulin (CD141), and vascular endothelial growth factor (VEGF)) have been proposed as potential risk factors and prognostic biomarkers of ND, such as cognitive impairment, Alzheimer’s disease, and PD [8,9,10,11,12,13,14,15,16,17,18]. Accordingly, recent evidence documents a significant association between endothelial dysfunction and the onset and progression of NDs, as well as between plasma levels of SDCs and NDs [8,9,10,11,12,13,14,19,20]. In line with such evidence, our group has recently conducted studies, which support the presence of higher levels of such SDC molecules, and particularly of SDC-1, SDC-2, and SDC-3, in AD subjects compared to the control group [17,18]. Certainly, such data are emerging and need further investigation in all NDs. For this purpose, in this study, we quantified the systemic levels of the four SDCs, ELA-54, ELA-21, and ELA-32, CD141, and VEGF in 30 subjects with PD compared to 30 matched healthy controls. Furthermore, we also wanted to determine whether significant levels of the molecules described above correlated with differences in the systemic levels of some blood biomarkers of peripheral inflammation and lipid metabolic disorder, including high-sensitivity C-reactive protein (hs-CRP), neutrophil/lymphocyte ratio (NLR), neutrophil/HDL-C ratio (NHR), and lymphocyte/HDL-C ratio (LHR), recognized to be involved in the pathogenesis of PD, as well as with the circulating levels of brain-derived neurotrophic factor (BDNF) involved in changes of neuroprotection and synaptic plasticity in NDs [10,11,21,22,23]. For assessing the key role of the molecules studied as risk factors, or rather as independent risk factors, univariate and multivariate regression analyses were performed, as well as correlations with clinical indicators, including the severity of PD expressed by the Hoehn and Yahr (H&Y) scale. Finally, receiver operating characteristic (ROC) curves were used to evaluate the diagnostic performance of the molecules investigated in PD. This allowed us to evaluate the relevance of these molecules as risk factors and diagnostic or prognostic biomarkers of PD and the possible necessity to combine them in a unique detection to facilitate a differential diagnosis and prognosis of the PD cases compared to the healthy control (HC) subjects.

## 2. Results

### 2.1. Comparison of the Baseline Demographic Characteristics and Laboratory Indices Between the PD Group and the HC Group

In Table 1, the comparisons of the baseline data between the HC and PD patients are reported. There were no significant differences in age and sex between the two groups (*p* = 0.27 and *p* = 0.29, *t*-test with Welch’s correction). In laboratory tests, the PD group had higher WBC count, neutrophil count, NLR ratio, TG, TC, and hs-CRP levels compared to the HC individuals, and the differences were statistically significant (*p* = 0.03, *p* = 0.0052, *p* = 0.0057, *p* = 0.0039, and *p* < 0.0001, respectively, *t*-test with Welch’s correction, see Table 1). Additionally, the PD group had slightly lower lymphocyte levels than the HC group, although this difference was not statistically significant (*p* = 0.39, *t*-test with Welch’s correction, see Table 1). However, they were significantly correlated with neutrophil levels (r = −0.14, *p* = 0.001, linear Pearson correlation test). The HDL-C levels were significantly higher in the HC group than in the PD cases, with a statistically significant difference (*p* < 0.0001, *t*-test with Welch’s correction, see Table 1). Accordingly, a statistically significant difference in the values of NHR and LHR between the two groups, HC and PD, was observed (*p* < 0.0001, *t*-test with Welch’s correction, see Table 1). Lastly, we observed a statistically significant difference in the plasma levels of BDNF between the two groups (*p* < 0.0001, *t*-test with Welch’s correction, see Table 1).

### 2.2. Comparisons of ELAs, SDCs, CD141, and VEGF Molecules Between the HC and PD Cases

**Figure 1:**
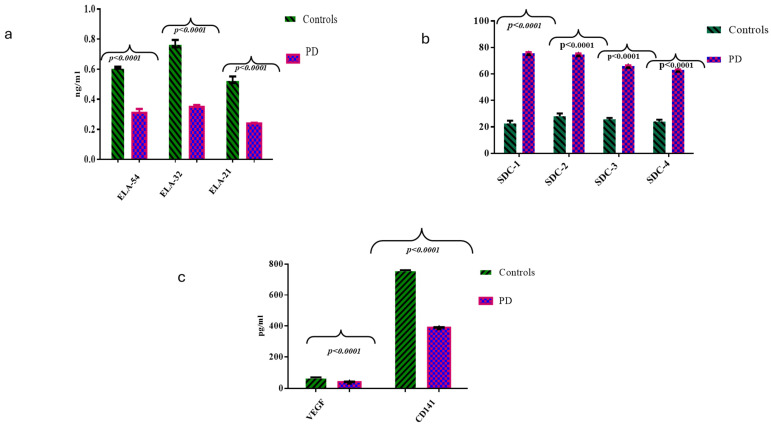
(**a**–**c**). Systemic levels of ELA peptides, shed SDC (1–4), VEGF, and CD141. In (**a**), it reports the mean values of the systemic levels of the molecules under study. Statistical analysis demonstrated, in subjects affected by PD, a significant reduction in the circulating levels of the peptide ELA-54 and its degradation products, ELA-32 and -21: in particular, in the PD group, ELA-21 was significantly lower than the other two peptides; in particular, subjects affected by PD showed ELA-21 levels equal to 0.24 ng/mL ± 0.4 ng/mL vs. 0.53 ng/mL ± 0.2 ng/mL in healthy elderly subjects (*p* < 0.0001, *t*-test analysis and Welch’s correction). Therefore, a significantly decreasing expression kinetics of the peptides described above characterized the cases than the controls. By analyzing the circulating levels of SDC-1–4 (see (**b**)) between the two cohorts included in the study, significantly higher levels of the four SDCs studied, and particularly of SDC-1 and -2, were found. Precisely, SDC-1 and -2 showed significantly higher systemic levels, compared to the other SDCs, in subjects with PD respect to the controls (75 ng/mL ± 0.96 ng/mL in PD cases vs. 28 ng/mL ± 2.2 ng/mL in the controls, and 74.8 ng/mL ± 2.1 ng/mL in controls, respectively, *p* < 0.0001; *t*-test analysis and Welch’s correction) (see (**b**)). Furthermore, we also detected higher levels of thrombomodulin (CD141) and a significant reduction of VEGF in PD than the other groups (390 ± 1.10 pg/mL in PD vs. 760 ± 1.28 pg/mL in controls; 40 ± 1.2 pg/mL in PD cases vs. 68 ± 1.81 in in controls; *p* < 0.0001; *t*-test analysis and Welch’s correction; see (**c**)).

As shown in Figure 1a–c, the analysis of data (*t*-test with Welch’s correction) revealed statistically significant differences between the two groups in the ELA-54 peptide and its degradation products, ELA-32 and ELA-21, with decreasing values of these molecules moving from the HC group (higher values) to PD (lower values) (*p* < 0.0001 for all the comparisons, see Figure 1a). Therefore, significantly decreased levels of the ELA peptides characterized the PD individuals rather than the controls. Interestingly, circulating levels of the 4 SDC molecules showed an opposite trend compared to that observed for ELAs. Specifically, the control subjects (both after *t*-test analysis and Welch’s correction) presented the lowest values for each SDC assessed compared to the highest values detected in the PD cases, and all these differences were statistically significant (*p* < 0.0001 for all the comparisons, see Figure 1b). Furthermore, we also detected a significant decreasing trend from the controls to the PD cases for the thrombomodulin (CD141) and VEGF levels (*p* ≤ 0.0001 for all the comparisons after *t*-test analysis and Welch’s correction, see Figure 1c).

### 2.3. Univariate and Multivariate Regression Analyses

In the univariate analysis illustrated in Table 2, we identified several blood biomarkers associated with PD, including neutrophil count, HDL, TG, TC, hs-CRP, NLR, NHR, LHR, and BDNF. Additionally, we also detected that ELAs, the 4 SDC molecules, CD141, and VEGF molecules intriguingly showed to be significantly associated with PD. After adjusting for confounding variables, such as HDL, TG, and TC, the multivariate regression model showed that NLR, hs-CRP, ELA-32, SDC-1, SDC-2, SDC-3, and BDNF were independent risk factors for PD, as reported in Table 3. Additionally, we observed that the SDC-1, SDC-2, and SDC-3 plasma levels correlated positively with the inflammatory blood biomarkers and disease severity (i.e., the H&Y scale) and negatively with ELA-32 and BDNF, as shown in Table 4. Thus, the trend towards increased systemic levels of these molecules reflects the increased dysfunction of eGCX and the BBB endothelium and appears to be influenced by the inflammatory burden.

### 2.4. Evaluation of NLR, hs-CRP, SDC-1, SDC-2, SDC-3, ELA-32, and BDNF as PD Biomarkers: ROC Curve Analyses

**Figure 2:**
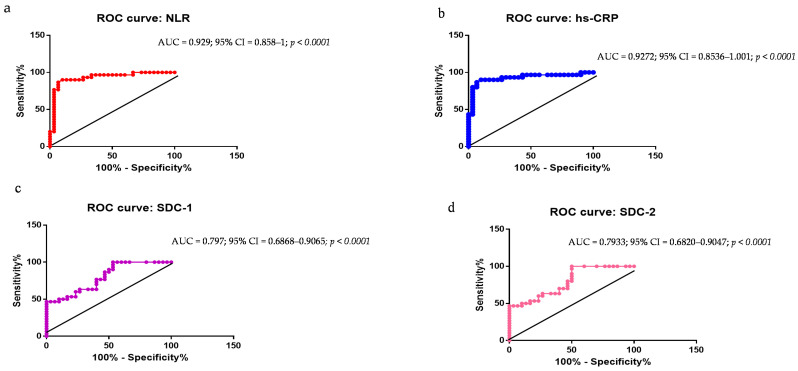

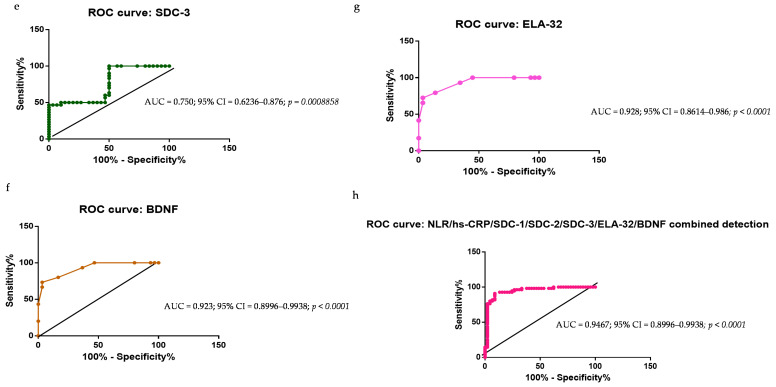
(**a**–**h**). ROC curves for evaluating the diagnostic performance of NLR, hs-CRP, SDC-1, SDC-2, and SDC-3, ELA-32, and BDNF as biomarkers. ROC curves were constructed for assessing the diagnostic performance of the abovementioned molecules able to distinguish patients with PD from HC. Statistically analyses showed significant data, specifically: (**a**) the area under the curve (AUC) of the ROC for distinguishing PD from the HCs based on NLR was 0.929 (95% CI, 0.858–1; *p* < 0.0001); (**b**) based on hs-CRP, the AUC was 0.9272 (95% CI, 0.853–1.001); (**c**) based on SDC-1, the AUC was 0.796 (95% CI, 0.686–0.906); (**d**) based on SDC-2, the AUC was 0.793 (95% CI, 0.682–0.904); (**e**) based on SDC-3, the AUC was 0.750 (95% CI, 0.623–0.874); (**f**) based on BDNF, the AUC was 0.923 (95% CI, 0.899–0.993); and (**g**) based on ELA-32, the AUC was 0.928 (95% CI, 0.861–0.986). However, such molecules did not have a statistically promising sensitivity and specificity in all the performed analyses (see (**a**–**g**), ROC curves). Accordingly, the data of all these molecules were combined in a unique detection for increasing their diagnostic performance and enabling a better distinction of PD cases from HC. The data, indeed, were particularly significant: AUC = 0.946 (95% CI, 0.899–0.993; *p* < 0.0001), with a sensitivity and specificity significantly improved, as shown in the ROC curve in (**h**).

ROC curves were constructed for evaluating the diagnostic performance of the abovementioned molecules able to distinguish patients with PD from HCs. The executed analyses statistically demonstrated significant data, specifically, (a) the area under the curve (AUC) of the ROC for distinguishing PD from the HCs based on NLR was 0.929 (95% CI, 0.858–1; *p* < 0.0001); (b) based on hs-CRP, the AUC was 0.9272 (95% CI, 0.853–1.001); (c) based on SDC-1, the AUC was 0.796 (95% CI, 0.686–0.906); (d) based on SDC-2, the AUC was 0.793 (95% CI, 0.682–0.904); (e) based on SDC-3, the AUC was 0.750 (95% CI, 0.623–0.874); (f) based on BDNF, the AUC was 0.923 (95% CI, 0.899–0.993); and (g) based on ELA-32, the AUC was 0.928 (95% CI, 0.861–0.986). However, we observed that such molecules did not have a statistically promising sensitivity and specificity in all the performed analyses (see Figure 2a–g, ROC curves). Accordingly, we combined the data of all these molecules in a unique detection for increasing their diagnostic performance and enabling a better distinction of PD cases from HCs. The data, indeed, were particularly significant: AUC = 0.946 (95% CI, 0.899–0.993; *p* < 0.0001), with a sensitivity and specificity significantly improved, as shown in the ROC curve in Figure 2h.

## 3. Discussion, limitations and suggested strategies 

PD is a progressive ND, and valid biomarkers are needed to improve early diagnosis and monitoring of prognosis and outcome [1]. The pS129-α-syn, which represents the predominant form of α-syn, observed in Lewy bodies (i.e., abnormal aggregations of proteins that develop inside neurons affected by PD), has been considered a promising diagnostic biomarker of PD. Higher levels of this molecule have been detected in PD cases compared to controls in many studies reported in the literature [24], although not statistically significantly in most of them [6]. Furthermore, many of these studies included only a small sample size.

Several molecules are emerging as potential PD biomarkers, although current data seem preliminary and need further validation. Among these, molecules and indices related to eGCX/endothelial dysfunction, systemic inflammation, modified lipid metabolism, and the alterations in neuroprotection and synaptic plasticity, appear promising. They show a close relationship with neuroinflammation and the onset of NDs, including PD [8,13,25,26]. Accordingly, our group has recently conducted studies, which support the presence of higher circulating levels of such molecules in AD subjects compared to the control group [17,18]. These promising data led us in this study to evaluate the role of such molecules in PD. Specifically, we first examined the eventual presence of statistically significant differences in systemic levels of various biochemical and hematological inflammatory biomarkers derived from routine blood tests or as ratios of these measurements. Among these, the analysis primarily included the absolute numbers of neutrophils and lymphocytes and their ratio, the NLR parameter, and the molecules related to lipid metabolism. Neutrophils and lymphocytes are crucial in the initiation and amplification of the systemic inflammatory response, as well as neuroinflammation [11,22,23]. Furthermore, NLR, as an easily computable laboratory biomarker, has been shown in several studies to have significant associations with the onset and progression of various diseases, including NDs [27,28]. Accordingly, a recent meta-analysis revealed that increased NLR levels are significantly associated with PD. However, further research is suggested for identifying the potential clinical benefits of this simple and inexpensive biomarker in the diagnosis of PD [29]. Additionally, Grillo’s group recently demonstrated in an in vivo study conducted on 61 PD cases and 60 age- and sex-matched controls that an increased NLR reflects protein changes in the α-syn and amyloid-β pathways associated with central neurodegeneration and increased clinical burden [30]. Here, we discovered that NLR, more than neutrophils, represents an independent risk factor for PD. The same result was obtained in our study by examining systemic hs-CRP levels. On the other hand, a recent meta-analysis conducted by a Chinese group [31] on twenty-three eligible case–control studies, involving 4598 individuals (2646 PD patients and 1932 healthy controls), displayed a significant association between increased systemic hs-CRP levels and PD. Similarly, we observed that the NHR and the LHR, related to lipid metabolism, were influencing risk factors for PD when compared with the systemic levels of the NHR and the LHR in the HC subjects. These data agree with recent meta-analyses reporting a dysregulated lipid metabolism in PD and dementia cases [32,33,34,35,36]. In addition, we also assessed significant differences in the systemic levels of TC and TG in PD cases compared to the HCs, demonstrating them to be influencing risk factors for PD but failing in such a potential when analyzed using the multiple regression model.

Interestingly, we also explored the role of BDNF in PD. BDNF is reported in the literature to be associated with PD neuropathology [11,37]. It regulates the survival of dopaminergic neurons in the substantia nigra and increases the functional activity of striatal neurons. Quantitative and functional declines in BDNF, as well as in its TrkB receptor, cause degeneration of dopaminergic neurons and accumulation of α-syn in the substantia nigra and neuroinflammation [11,37,38]. Accordingly, exogenous treatment with BDNF has been recently suggested by Ebert’s group to promote neuro-regeneration in NDs [39]. Recent evidence has underlined that BDNF/TrkB signaling is decreased in PD and linked to disease severity and long-term complications [37]. Accordingly, we observed higher levels of BDNF in the HC individuals than in the PD cases, which significantly correlated with PD severity. Multivariate regression also demonstrated that BDNF was an independent PD risk factor. Additionally, we examined the diagnostic potential of this molecule by observing significant data.

The alterations detected in the systemic levels of both inflammatory and neuroprotective molecules examined, such as BDNF, have been documented in some studies to be significantly associated with the BBB and cerebrovascular unit dysfunction [11,15,37,38]. Specifically, they contribute to determining eGCX and endothelial dysfunction [8,11,13,17,18]. Considering this interesting evidence, we also evaluated the levels of four SDCs, CD141, VEGF, and ELA peptides in our study population. Significant differences in the systemic levels of such molecules were measured. In addition, SDC-1, SDC-2, and SDC-3, ELA-32, VEGF, and CD141 also proved to be both influencing and independent risk factors of PD. Furthermore, their levels were observed to correlate positively with the systemic levels of hs-CRP, neutrophils, and NLR and negatively with the systemic reduced levels of BDNF and ELA-32, both having regulative functions in the homeostasis of the brain. Such data also agree with the literature evidence of the close relationship between SDCs and the accumulation in the brain of α-syn [39,40,41,42]. A positive correlation was also assessed between the increase in SDC-1, SDC-2, and SDC-3, ELA-32, VEGF, and CD141 levels and PD severity, evaluated using the disability H&Y scale. These data emphasize the close relationship of the alterations in both the blood–brain barrier and the vascular unit with the onset and progression of NDs, such as PD.

Such promising results on the molecules described above led us to explore their diagnostic value as biomarkers of PD. Consequently, ROC curve analyses were used to evaluate the diagnostic efficacy of these molecules and whether they significantly facilitated the distinction between the subjects with PD and the HCs. The results obtained were promising, with significant AUC values > 0.5, particularly when we performed a combined detection. This suggests that the diagnostic performance of such molecules increases by combining them in a unique panel, and probably also by adding the levels of α-syn in further studies.

### 3.1. Limitations

This pilot study provides preliminary evidence supporting the diagnostic and prognostic potential of a panel of blood-based biomarkers in PD. However, several limitations must be recognized to contextualize the obtained findings and guide future research. The main limitation of the study is the modest sample size, which included only 60 participants. Small sample sizes are not unusual in pilot studies, but they may limit the statistical power of the results. Moreover, the PD cohort included patients with different grades of disease severity, but the small sample size precluded the subgroup analyses to explore potential differences in biomarker profiles across diverse stages of pathology. Further, the NLR and hs-CRP, as biomarkers of systemic inflammation, could be easily influenced by subclinical conditions, lifestyle factors, or comorbidities, by reducing the specificity of these biomarkers for PD. Furthermore, the study relied solely on plasma samples, without exploring other biological matrices such as cerebrospinal fluid (CSF) or brain tissue, which may provide more direct insights into PD pathophysiology and neuroinflammation. Finally, the study did not employ advanced omics approaches (e.g., proteomics, metabolomics) to better profile the molecular landscape of PD. Such approaches would also be encouraged [43,44,45] as they might facilitate both the validation and confirmation of the role of the molecules investigated in our study, as well as suggest them as appropriate biomarkers towards personalized management [46,47,48].

### 3.2. Strategies to Address Limitations

To overcome these limitations and strengthen the evidence for the proposed biomarkers, we suggest primarily increasing the sample size for improving statistical power, ensuring the broader representation of PD patients, including diverse demographic, clinical, and biochemical profiles, and allowing differential management for diverse stages of disease, as well as for several PD subgroups. To enhance the specificity of the NLR and hs-CRP, as well as all the other molecules suggested in the combined profile, a further strengthening of the exclusion criteria through questionnaires and clinical evaluations could be useful for future studies to adjust the confounding variables [49,50]. The validation of biomarker levels in CSF would provide a more comprehensive understanding of their relevance to PD pathophysiology, and post-mortem brain tissue analysis could further confirm the role of endothelial glycocalyx dysfunction and neuroinflammation in PD. Further, the application of multi-omics approaches could uncover novel biomarkers, e.g., miRNA and pathways associated with PD [51]. In addition, as a future suggestion, given the promising role of BDNF and ELA-32 as neuroprotective molecules, preclinical and clinical studies should investigate their therapeutic potential. For instance, BDNF-based therapies or ELA peptide analogs could be tested in animal models of PD to assess their ability to alleviate neuroinflammation and neuronal loss. Similarly, interventions targeting endothelial glycocalyx integrity could be explored to reduce syndecan shedding and vascular dysfunction.

## 4. Population and Methods

### 4.1. Population Enrolled

A cohort of 60 subjects, 30 patients affected by PD (age range, 60–81 years; mean age, 71.2 ± 4.8 years; 11 females and 19 males) and 30 age- and sex-matched controls (age range, 66–83 years; mean age, 71.8 ± 2.8 years; 15 males and 15 females), was included in the present study. PD patients admitted to the Department of Biomedicine, Neuroscience, and Advanced Diagnostics, Affiliated Hospital “Paolo Giaccone” of the University of Palermo between January 2023 and March 2024 were enrolled in this study, according to the diagnostic criteria established by the United Kingdom Parkinson’s Disease Society Brain Bank [52]. In addition, the following exclusion criteria were considered: (1) severe hepatic or renal impairment; (2) history of immunosuppression or anti-inflammatory treatment; (3) acute infection; (4) autoimmune disease; (5) significant clinical emotional disturbances, resulting in an inability to cooperate with clinical evaluation. The age- and sex-matched healthy control group (HC) consisted of healthy subjects and was recruited from the health examination center of our hospital during the same period. The study was approved by the Ethics Committee at the Affiliated Hospital of the University of Palermo (2023-UP-00346).

### 4.2. Clinical Participants’ Information Collection

Baseline information and laboratory data, mainly including routine blood tests and blood biochemistry results, were collected from all the enrolled subjects. Based on the Hoehn–Yahr (HY) scale, 8 patients were classified as early-stage (1–2.5; namely, 7 with HY 2 and 1 with HY 2.5) and 22 as intermediate-to-late-stage (≥3). In addition, we calculated the following parameters for each patient and HC: neutrophil/lymphocyte ratio (NLR), neutrophil/HDL-C ratio (NHR), and lymphocyte/HDL-C ratio (LHR) for each patient and HC, where NLR = absolute neutrophil count divided by the absolute lymphocyte count; NHR = absolute neutrophil count divided by the HDL lipoprotein amount; LHR = absolute lymphocyte count divided by the HDL lipoprotein amount.

### 4.3. Quantification of Plasma Levels of hs-CRP, BDNF, ELAs, SDCs, CD141, and VEGF Molecules

Venous blood samples were collected from all the enrolled subjects in a fasting state (>8 h without food intake). Plasma samples were obtained immediately after collection by centrifuging whole blood (3500 rpm at 4 °C for 10 min) and then stored at −80 °C for further analysis. Plasma levels of ELA-54, ELA-32, and ELA-21 were determined using a commercial human ELISA kit (BMA BIOMEDICALS, Augst, Switzerland). Plasma levels of SDC-1, SDC-2, SDC-3, and SDC-4 were detected by using commercial ELISA kits (SDC-1: Bio-Techne Ltd., Abingdon, UK; SDC-2 and SDC-3: My BioSource, San Diego, CA, USA; SDC-4: R&D Systems, Minneapolis, MN, USA). Furthermore, thrombomodulin (CD141) and VEGF plasma levels were quantified by using commercial ELISA kits (Bio-Techne Ltd., Abingdon, UK), while hs-CRP measurements were conducted with a Cobas Integra analyzer (Roche Diagnostics, Penzberg, Germany) using the turbidimetric method. The reference range for hs-CRP was <0.5 mg/L. Moreover, the quantification of plasma BDNF levels was performed using a commercial human ELISA kit (CD_Creative Diagnostics, Shirley, NY, USA). The operating procedures for all the molecules evaluated were carried out according to the manufacturer’s instructions.

### 4.4. Statistical Analyses

Concerning statistics, the results obtained were analyzed using R version 4.3.3 (The R Foundation) and GraphPad Prism 10 software version 10.4.2 (GraphPad Software, La Jolla, CA, USA). All *p*-values were two-sided, and those below 0.05 were considered statistically significant. Mean values with SDs were calculated for quantitative variables due to a normal distribution. Differences between the two groups were evaluated using the Kolmogorov–Smirnov test or Student’s *t*-test when appropriate, while one-way ANOVA or Kruskal–Wallis tests followed by Bonferroni correction were applied to compare more than two groups. Differences between the non-quantitative variables of the two groups, HC and PD, were calculated using the chi-squared test (table 2 × 2). The correlations between the two continuous variables were assessed with Pearson’s test or the non-parametric Spearman correlation test. Further, smooth curve fitting was employed to describe the relationship between variables and PD. To detect the diagnostic performance of the investigated molecules in PD, ROC curves were generated.

## 5. Conclusions

In summary, we cannot accurately draw effective conclusions. However, we can suggest that these molecules seem promising in facilitating the complex management of PD. Our data support this evidence for the first time and seem to shed light on more specific PD biomarkers than α-syn. The combined use of these biomarkers in a panel demonstrated a superior diagnostic performance compared to individual biomarkers, suggesting their utility in facilitating early diagnosis and personalized management of PD, including assessment and prediction. Identifying risk factors and biomarkers for PD may clarify disease mechanisms, facilitate the identification of molecules as biomarkers and therapy targets, as well as improve prevention, diagnosis, prognosis, treatment, and post-treatment management.

## Figures and Tables

**Table 1:** Demographic and clinical characteristics of PD patients and the healthy controls (HCs).

Demographic and Clinical Variables	HCs (N = 30)	PD (N = 30)	*p*-Value
**Age**	71.8 ± 2.8	71.2 ± 4.8	0.27 *
**Gender (** **N, %)** **Male** **Female**	15 (50%) 15 (50%)	19 (63%) 11 (37%)	0.29 **
**WBC (×10** **^9^** **/L)**	5.6 ± 1.4	6.7 ± 2.88	0.03 *
**Lymphocytes (×10** **^9^** **/L)**	1.8 ± 2.2	1.63 ± 2.7	0.39 *
**Neutrophils**	3.3 ± 1.18	4.4 ± 1.9	0.0052 *
**HDL-C (mg/dL)**	55 ± 0.48	52 ± 0.29	<0.0001 *
**TG** **TC**	1.6 ± 0.71 4.8 ± 1.0	1.11 ± 0.52 4.1 ± 1.02	0.0057 * 0.0039 *
**hs-CRP (mg/L)**	2.4 ± 2.9	8.8 ± 6.9	<0.0001 *
**NLR**	1.9 ± 1.3	2.7 ± 1.7	0.016 *
**NHR** **LHR**	54.4 ± 2.9 34.4 ± 1.4	59.95 ± 3.1 36.7 ± 2.6	<0.0001 * <0.0001 *
**BDNF (pg/mL)**	8.2 ± 1.2	4.5 ± 2.2	<0.0001 *
**H&Y**	N/A	2.8 ± 1.9 (2–4)	-

Note: HDL-C, high-density lipoprotein cholesterol; TG, triglyceride; TC, total cholesterol; hs-CRP, high-sensitivity C-reactive protein; NLR, neutrophil–lymphocyte ratio; NHR, absolute neutrophil count divided by the amount of HDL lipoproteins; LHR, absolute lymphocyte count divided by the amount of HDL lipoproteins; H&Y, Hoehn and Yahr scale; * *p*-values calculated using the *t*-test with Welch’s correction; ** *p*-values calculated using the chi-squared test (table 2 × 2).

**Table 2:** Factors influencing the analysis of PD.

Variables	OR (95%CI)	*p*-Value
Neutrophils	5.2 (1.28–10.6)	0.0051
**HDL-C**	4.2 (2.23–7.8)	**0.0001**
**TG**	3.2 (1.27–8.9)	**0.0003**
TC	3.3 (1.45–6.9)	0.0048
**hs-CRP**	10.3 (0.23–19.8)	**<0.0001**
**NLR**	6.2 (1.28–10.6)	**0.0003**
**NHR**	9.5 (1.23–16.8)	**<0.0001**
**LHR**	7.9 (1.23–9.6)	**0.0001**
**BDNF**	7.5 (0.26–10.1)	**0.0001**
ELA-32	2.5 (1.56–7.2)	0.003
**SDC-1**	3.1 (3.7–6.9)	**0.0001**
**SDC-2**	4.1 (2.3–7.9)	**0.0004**
**SDC-3**	5.1 (2.3–7.9)	**0.0001**
CD141	2.5 (4.3–8.7)	0.02
VEGF	3.9 (8.7–11.3)	0.045

Note: HDL-C, high-density lipoprotein cholesterol; TG, triglyceride; TC, total cholesterol; hs-CRP, high-sensitivity C-reactive protein; ELA, Elabela peptide; NLR, neutrophil–lymphocyte ratio; NHR, absolute neutrophil count divided by the amount of HDL lipoproteins; LHR, absolute lymphocyte count divided by the amount of HDL lipoproteins; SDC, syndecan; VEGF, vascular endothelial growth factor; CD141, thrombomodulin.

**Table 3:** Multivariate regression analyses for PD.

Variables	OR (95%CI)	*p*-Value
**hs-CRP**	9.7 (0.29–15.8)	**0.0001**
NLR	4.1 (1.23–9.1)	0.003
**BDNF**	6.9 (0.25–9.1)	**0.0001**
ELA-32	2.5 (1.56–7.2)	0.003
**SDC-1**	5.1 (3.7–6.9)	**0.0001**
**SDC-2**	4.1 (2.3–7.9)	**0.0003**
**SDC-3**	5.9 (2.6–9.7)	**0.0001**

Note: hs-CRP, high-sensitivity C-reactive protein; NLR, neutrophil–lymphocyte ratio; SDC, syndecan; BDNF, brain-derived neurotrophic factor; ELA, Elabela peptide.

**Table 4:** Correlations of SDC-1, SDC-2, and SDC-3 plasma levels with inflammatory blood biomarkers, ELA-32, BDNF, and PD severity.

Variables	β	*p*-Value *
**hs-CRP**	0.16	0.0001
**Neutrophils**	0.19	0.0002
**NLR**	0.22	0.003
**ELA-32**	−0.24	0.004
**BDNF**	−0.17	0.0001
**H&Y scale**	0.15	0.0001

Note: hs-CRP, high-sensitivity C-reactive protein; NLR, neutrophil–lymphocyte ratio; SDC, syndecan; BDNF, brain-derived neurotrophic factor; ELA, Elabela peptide; H&Y, Hoehn and Yahr scale. * *p* values were calculated with with Pearson’s test or the non-parametric Spearman correlation test.

## Data Availability

All relevant data are within the paper.
